# Ten years of simulation-based shoulder dystocia training- impact on obstetric outcome, clinical management, staff confidence, and the pedagogical practice - a time series study

**DOI:** 10.1186/s12884-018-2001-0

**Published:** 2018-09-05

**Authors:** Johanna Dahlberg, Marie Nelson, Madeleine Abrandt Dahlgren, Marie Blomberg

**Affiliations:** 1 Department of Clinical and Experimental Medicine, Linköping University, Linköping, Sweden; 2 Department of Obstetrics and Gynecology, Linköping University, Linköping, Sweden; 3 Department of Medicine and Health Sciences, Linköping University, Linköping, Sweden

**Keywords:** Team training, Simulation, Debriefing, Shoulder dystocia, Brachial plexus injury

## Abstract

**Background:**

To assess the impact of 10 years of simulation-based shoulder dystocia training on clinical outcomes, staff confidence, management, and to scrutinize the characteristics of the pedagogical practice of the simulation training.

**Methods:**

In 2008, a simulation-based team-training program (PROBE) was introduced at a medium sized delivery unit in Linköping, Sweden. Data concerning maternal characteristics, management, and obstetric outcomes was compared between three groups; prePROBE (before PROBE was introduced, 2004–2007), early postPROBE (2008–2011) and late postPROBE (2012–2015). Staff responded to an electronic questionnaire, which included questions about self-confidence and perceived sense of security in acute obstetrical situations. Empirical data from the pedagogical practice was gathered through observational field notes of video-recordings of maternity care teams participating in simulation exercises and was further analyzed using collaborative video analysis.

**Results:**

The number of diagnosed shoulder dystocia increased from 0.9/1000 prePROBE to 1.8 and 2.5/1000 postPROBE. There were no differences in maternal characteristics between the groups. The rate of brachial plexus injuries in deliveries complicated with shoulder dystocia was 73% prePROBE compared to 17% in the late postPROBE group (*p* > 0.05). The dominant maneuver to solve the shoulder dystocia changed from posterior arm extraction to internal rotation of the anterior shoulder between the pre and postPROBE groups. The staff questionnaire showed how the majority of the staff (48–62%) felt more confident when handling a shoulder dystocia after PROBE training. A model of facilitating relational reflection adopted seems to provide ways of keeping the collaboration and learning in the interprofessional team clearly focused.

**Conclusions:**

To introduce and sustain a shoulder dystocia training program for delivery staff improved clinical outcome. The impaired management and outcome of this rare, emergent and unexpectedly event might be explained by the learning effect in the debriefing model, clearly focused on the team and related to daily clinical practice.

**Electronic supplementary material:**

The online version of this article (10.1186/s12884-018-2001-0) contains supplementary material, which is available to authorized users.

## Background

Emergent obstetrical events are rare but will always occur. It is critical for staff to be well trained and coordinated and confident when a complication appears, even when it happens so seldom that staff members will not gain experience by frequent clinical work. One way to tackle the dilemma is to introduce different types of skills and simulation training programs for the staff team. Research evidence supports that team training and simulation exercises improve technical skills, team communication, coordination, and documentation [1–3]. It is also well described how staff confidence increases after team-based obstetric skills and simulation exercises [4–6]. When it comes to improved obstetric and neonatal outcomes, only a small percentage of reports primarily addresses clinical outcomes and results are inconsistent [7]. One multicenter randomized control trial from the Netherlands showed how a one-day, off-site, simulation-based team training did not reduce obstetric complications (low Apgar score, severe postpartum hemorrhage, trauma due to shoulder dystocia, eclampsia, hypoxic ischemic encephalopathy) [8]. In contrast an Australian retrospective cohort study reported an overall improvement in some clinical outcomes (Apgar at 1 minute, umbilical cord lactate, and average length of infant’s stay in clinic) after a single day of training for the trainers at eight different maternity units [9]. Crofts et al. found significant benefits of a long-term multiprofessional 1-day training course with improvements in clinical outcomes, including the prevalence of brachial plexus injury due to shoulder dystocia [10]. Comparable results concerning a decrease in obstetric brachial plexus injury was found after introduction of a shoulder dystocia training protocol [11, 12]. Others could not show a beneficial effect on the number of children injured due to shoulder dystocia after introducing simulation-based team training [13, 14].

When reflecting on the diverse picture of the impact of simulation training on clinical outcome previous studies share the fact that they do not provide detailed information of contextual factors and how the training was performed. This makes it difficult to compare and understand what pedagogical arrangements that support improvement of clinical outcomes. In general, simulation training traditionally follows a pedagogical model comprising three phases. In the introductory briefing phase, the participants are introduced to the scenario and receive information about the situation they are going to encounter as they start the simulation. The following phase is the practical enactments of the simulation, where the team members act in their professional role in collaboration to take care of the patient in the emerging situation. The final phase is the debriefing where the process and outcomes of the simulated scenario is discussed [15]. From a learning perspective, debriefing sessions are considered central for the participants’ learning and provide the learner with the opportunity to reflect their understanding of the course of the scenario and their actions and interactions in the team [16]. However, the achieved level of reflection is interconnected with the questions posed by the facilitator and it is a challenging task for the instructor to facilitate reflection at a deep level in the participants [17]. It is reasonable to assume that different approaches to pedagogy in simulation also might influence clinical outcomes differently.

The aims of the present study were to evaluate the impact of 10 years of simulation-based shoulder dystocia training on clinical outcomes for the mother and the infant, on staff confidence, on management in the delivery room, and to scrutinize the characteristics of the pedagogical practice of the simulation training.

## Methods

The present study applies a mixed method approach, combining quantitative obstetric outcome data with qualitative analyses of video-recorded simulation based training sessions. The use of a mixed methods research approach was claimed to gain a deeper and broader understanding than using only one research design, and to allow for contextualizing information that might provide richer insights into the phenomenon under study [18].

In a mixed methods approach, quantitative and qualitative data can be integrated through different study designs, methods and interpretations [19]. In the present study, an explanatory sequential design was applied, meaning that the researchers first collected and analyzed the quantitative data, and these data then informed the qualitative data collection and analysis. In our findings, we are presenting the quantitative and qualitative data in different but contiguous sections, in an attempt at integrating clinical outcomes with contextual factors and thereby develop a complementary picture, that examine process experiences along with outcomes [20].

### The simulation program

In 2008, a simulation-based team-training program, Practical obstetric team-training (PROBE), was introduced in the delivery ward at the University Hospital, Linköping, Sweden. The objectives were to improve obstetric emergency skills and develop interprofessional teamwork, thus promoting better patient outcome. Participation in PROBE was mandatory for obstetricians, midwifes and nurse assistants who worked at the delivery ward. Participants were scheduled during working hours for simulation exercises at an interval of 1.5 years. To cover the need of staff’s regular training, PROBE was organized six times each year. The PROBE sessions took place at the clinical training center, Clinicum, at the University Hospital, a simulation center equipped with an obstetric skills laboratory. Each team session was scheduled for 3 hours, including two simulation scenarios and one practical skills training station. During the whole study period, PROBE included a 40-min shoulder dystocia scenario. Obstetric emergencies were simulated using actors, usually instructors, and/or mannequins, depending on the scenario.

All participants were prepared in advance by individually studying a theoretical management course specific for the complications to be trained. In each simulation, staff members worked in maternity care teams of one or two midwifes, one doctor, and one nurse assistant so that the simulations would be as realistic as possible.

The PROBE program is based on clinical skills suggested by Advanced Life Support in Obstetrics (ALSO®^)^, but has been further developed since PROBE also had focus on team work and communication. Each scenario was led by certified and experienced instructors, both midwifes and obstetricians, who had participated in ALSO® training courses [21]. The aim of the simulation exercises was for the participants to recognize an emergency and use the subsequent standardized management methods and procedures established in the obstetric department’s clinical guidelines. The mnemonic to remember in managing shoulder dystocia was HELPERR (H— call for Help, E—consider Episiotomy, Legs—McRobert’s maneuver, Pressure—Suprapubic pressure externally, Enter—Enter the vagina using internal pressure to reduce impacted shoulder, finally using a Wood’s screw maneuver to bring the shoulders into oblique diameter and 180 degrees rotation, and, if necessary, Remove—the posterior arm. Finally, the last R—Rotate the patient to her hands and knees.)

Instructors observed the team and made notes during the whole simulation. Immediately after each scenario, the team along with the instructors reflected upon how the team had performed. The reflection consisted of three parts: first, everyone reconfirmed what happened chronologically, thereafter everyone stated what they did well, and, finally, everyone summarized what they had learned and would bring to clinical practice. Questions and the notes of the instructor were used to support the discussion.

### Quantitative data collection and statistics

To identify deliveries complicated with shoulder dystocia, all medical records with the ICD-10 diagnosis O66.0, obstructed labor due to shoulder dystocia, were extracted from the hospitals digital birth- and maternity care registration system Obstetrix® over the study period 2004–2015. Data were divided into three groups; prePROBE (before PROBE was introduced, 2004–2007), early postPROBE (2008–2011) and late postPROBE (2012–2015). Data concerning maternal characteristics, management at delivery, and obstetric and neonatal clinical outcomes were collected and compared between the prePROBE and the two postPROBE groups. Data collected were maternal age, parity, body mass index (BMI), incidence of diabetes mellitus, gestational weight gain (defined as maternal weight at delivery minus maternal weight at booking gestational week 8–10), gestational age at delivery, if labor was induced, if contractions were augmented with oxytocin, delivery method (normal vaginal delivery or vacuum extraction), and the prevalence of acute anal sphincter injury. Additional management variables at delivery were extracted, when the shoulder dystocia was confirmed, according to HELPPER; whether oxytocin infusion was stopped, if internal rotation of the anterior shoulder was performed, and/or if posterior arm extraction was done before the delivery.

Three times during the postPROBE period (year 2011, 2013 and 2016) staff who had attended PROBE-sessions responded to an electronic questionnaire (Additional file 1), which included questions about self-confidence and perceived sense of security in acute obstetrical situations, specifically obstetric emergency situations including shoulder dystocia trained at PROBE. Only staff members who had attended PROBE received the questionnaire.

Continuous variables are presented as mean value and standard error of the mean (SEM). Means in the three groups were compared using an analysis of variance, and a *p*-value for similarity was given. Categorical variables are presented as numbers and frequencies. Group differences between prePROBE and postPROBE groups were for categorical variables analyzed by Fischer exact test using StatXact, Cytel software corp.

### Qualitative data collection and analysis

For the purpose of this study, three maternity care teams participating in simulation exercises according to PROBE were video-recorded, both during the simulation exercise and the debriefing session. Empirical data from the pedagogical practice was gathered through observational field notes of the three video-recordings. The recordings and field notes were analyzed by the authors using collaborative video analysis [22]. Individual field notes were compared and consensus was negotiated through a constant comparative analysis [23]. Focus for the analysis was how the team members reconstructed the course of communication, individual actions and interactions in the team during the debriefing of the scenario. The researchers each contributed different professional background in healthcare work and in education, specifically medical education.

The Regional Ethical Review Board in Linköping, Sweden approved this study (Dnr 2016/177–31).

## Results

### Quantitative data

During the study period 2004–2015, there were 33,853 births in total distributed equally over the years, meaning about 3000 births each year. The number of non-instrumental vaginal deliveries increased from 73.8 to 82.4%, while the frequency of caesarean deliveries decreased from 17.0 to 11.3% (Table1). These dramatic changes in obstetric clinical practice could be explained by implementation of a nine-item list for organizational and cultural change, where PROBE is one of the nine items [24]. Among the deliveries, there was a statistically significant increase of diagnosed shoulder dystocia between the prePROBE and the early and late postPROBE periods, 0.9/1000 deliveries prePROBE compared to 1.8 and 2.5/1000 postPROBE (Table 1).

**Table 1:** The obstetric context during the study period, including all deliveries

	PrePROBE (2004–2007)	Early postPROBE (2008–2011)	Late postPROBE (2012–2015)	*p*-value
Number of deliveries	11,064	11,122	11,667	
Induction of labor, n (%)	1248 (11.3)	1411 (12.7)	1781 (15.2)	< 0.001
Non-instrumental vaginal delivery, n (%)	8169 (73.8)	8785 (79.0)	9620 (82.4)	< 0.001
Cesarean section, n (%)	1881 (17.0)	1560 (14.0)	1322 (11.3)	< 0.001
Vacuum extraction, n (%)	1014 (9.2)	777 (7.0)	725 (6.2)	< 0.001
Anal sphincter injury, n (%)	401 (3.6)	286 (2.6)	278 (2.4)	< 0.001
Number of shoulder dystocia, n (per 1000 deliveries)	11 (1.0)	20 (1.8)	29 (2.5)	0.005

There were no significant differences in maternal age, parity, BMI, gestational weight gain, or prevalence of diabetes mellitus type 1 between deliveries complicated with shoulder dystocia in prePROBE compared to the postPROBE groups (Table 2).

**Table 2:** Maternal and pregnancy characteristics in deliveries complicated with shoulder dystocia

	PrePROBE (2004–2007) *n* = 11	Early postPROBE (2008–2011) *n* = 20	Late postPROBE (2012–2015) *n* = 29	*p*-value
Maternal age, mean (SEM)	29.8 (1.64)	31.9 (1.28)	30.4 (1.23)	0.60
Primiparous, n (%)	5 (45)	9 (45)	10 (34)	0.45
BMI, mean (SEM)	27.8 (1.64)	27.7 (1.39)	27.7 (1.10)	0.99
Diabetes mellitus type 1, n (%)	3 (27)	1 (5)	3 (10)	0.27
Gestational weight gain (kg), mean (SEM)	13.0 (1.50)	15.2 (1.19)	14.8 (1.22)	0.49
Gestational age at delivery (days), mean (SEM)	278 (2.15)	285 (1.84)	282 (1.60)	0.15

*BMI* Body Mass Index

*SEM* Standard error of the mean

No significant differences were found concerning induction of labor and the use of oxytocin infusion between the prePROBE and the postPROBE groups (Table 3).

**Table 3:** Obstetric interventions and complications in deliveries with diagnosed shoulder dystocia

	PrePROBE (2004–2007) *n* = 11	Early postPROBE (2008–2011) *n* = 20	Late postPROBE (2012–2015) *n* = 29	*p*-value
Induction of labor, n (%)	4 (36)	3 (15)	9 (31)	0.94
Labor arrest, n (%)	4 (36)	12 (60)	19 (66)	0.12
Oxytocin infusion, n (%)	9 (82)	14 (70)	22 (76)	0.85
Instrumental delivery, n (%)	6 (54)	7 (35)	5 (17)	0.03
Anal sphincter injury n (%)	2 (18)	5 (25)	5 (17)	0.80

Fewer infants diagnosed with a shoulder dystocia were delivered instrumentally in the postPROBE group compared with the prePROBE group. The risk of anal sphincter injury was similar before and after the introduction of PROBE training.

No significant difference between the groups was observed concerning Apgar score or umbilical artery pH (Table 4).

**Table 4:** Infant outcomes in deliveries complicated with shoulder dystocia

	PrePROBE (2004–2007) *n* = 11	Early postPROBE (2008–2011) *n* = 20	Late postPROBE (2012–2015) *n* = 29	*p*-value
Umbilical artery pH, mean	7.17	7.20	7.20	
Apgar score < 4 at 1 min, n (%)	2 (18)	7 (35)	9 (31)	0.56
Apgar score < 7 at 5 min, n (%)	1 (9)	6 (30)	8 (27)	0.33
No brachial plexus injury or fracture, n (%)	2 (18)	10 (50)	20 (69)	0.005
Brachial plexus injury at birth, n (%)	8 (73)	8 (40)	5 (17)	0.001
Fractured clavicle, n (%)	1 (9)	2 (10)	2 (7)	0.76
Fractured humerus, n (%)	1 (9)	3 (15)	2 (7)	0.65
Early neonatal death	0	0	1	
Brachial plexus injury at 6 months follow up, n (%)	1 (9)	1 (5)	2 (7)	0.89

There was a significant decrease of infants born with a brachial plexus injury, or fracture of the clavicle or humerus, after the staff had attended PROBE training (Table 4). The rate of brachial plexus injuries in deliveries complicated with shoulder dystocia was 73% prePROBE compared to 17% in the late postPROBE group (Table 4), which was a significant reduction. There were a couple of persistent brachial plexus injuries in each group at 6 months of age.

The documentation in the medical records concerning maneuvers used after diagnosis of shoulder dystocia was significantly improved after PROBE training. When a shoulder dystocia is confirmed, the oxytocin infusion (if present) should be stopped, as further uterine contractions aggravate the entrapment of the infant. The oxytocin infusion was stopped significantly more often postPROBE compared to prePROBE (Table 5).

**Table 5:** Shoulder dystocia management before and after introduction of PROBE

	PrePROBE (2004–2007) *n* = 11	Early postPROBE (2008–2011) *n* = 20	Late postPROBE (2012–2015) *n* = 29	*p*-value
Management well documented, n	7	20	27	0.03
Stop of oxytocin infusion, n (%)	0/9 (0)	4/10 (40)	12/22 (54)	0.007
Internal rotation of the anterior shoulder, n (%)	1 (9)	8 (40)	15 (52)	0.06^a^
Posterior arm extraction performed, n (%)	6 (54)	12 (60)	13 (45)	
Data on maneuver missing	4	0	1	

^a^Internal rotation versus posterior arm extraction

Internal rotation of the anterior shoulder was used in 54% of the deliveries postPROBE compared to 9% prePROBE, a significant favor over time of this maneuver instead of posterior arm extraction.

The results of the staff questionnaire showed how the majority of the staff (48–62%) felt more confident when a shoulder dystocia occurred at the delivery unit after they had participated in PROBE training. The rest, 32–39%, answered that they had never experienced shoulder dystocia in clinical practice (Table 6). The response rate was between 68 and 78%.

**Table 6:** Questionnaire-based follow up of staff confidence after attending PROBE training

Year	Response rate	Do you feel more confident in your daily practice managing an *obstetric emergency situation* after PROBE training?	Do you feel more confident in your daily practice managing a *shoulder dystocia* after PROBE training?		
	N/total (%)	Yes, n (%)	Yes, n (%)	No, n (%)	I have never been in the situation, n (%)
2011	52/67 (78)	48 (94)	26 (51)	5 (10)	20 (39)
2013	62/91 (68)	55 (95)	28 (48)	9 (15)	22 (37)
2016	63/90 (70)	59 (94)	39 (62)	4 (6)	20 (32)

### Qualitative data

The collaborative video analysis showed that attention was drawn to how the facilitators/instructors support team reflections, and still supporting individual reflection for learning. All through the reflection, the experience from the simulation exercise was also related to clinical practice at the delivery unit. For instance, as an obstetrician stated “-Being summoned to a delivery room, not knowing what to expect, the need of communication and time to reflect on this information is crucial” Even if the team in the room already was aware of the next step, the obstetrician in charge has to consider different options based on information provided, before making final decisions and share this with the team.

Interestingly, as a first phase of the reflection, each of the team members was invited in sequence to describe when and how they acted and interacted during the scenario. The emerging narrative was told from each individual perspective, but grew into a web as all individuals in the team also included how they related to each other, building the bigger picture in their narrative. The instructor utilized the notes taken during the observation of the simulation exercise to recall and describe a particular sequence of events, in which every single activity was integrated. This thorough recapitulation made the team aware of every step in the process and every individual confident to speak up and contribute to the collective narrative. The team reflections were also guided and facilitated by probing questions that recalled the emerging collaboration and actions, such as “In what sequence did things happen?”, “Do everyone agree to this description?”, “What help did you need?”, “What happened when you arrived?”, or “Who decided what to do next?”. One example is when the obstetrician emphasized the value of the report from the coordinating midwife, and subsequently how the team agreed on the next action, and finally who would deliver the child.

In the second phase of the reflection, the instructor supported the identification of what each member did well and how this contributed to the teamwork. This is a technique that is commonly used in simulation-based training, but the typical feature of this phase in this case is that the instructors were building confidence in the participants through acknowledging that appreciating one’s own actions as knowing in practice that can be difficult to verbalize. One example is when the importance and value of continuous documentation of the process was emphasized when the actions of the assisting nurse were discussed as being responsible for documentation and the technical support. Confidence building was also discernible in relation to the performance of the whole team; e.g., when the two midwifes in the scenario discussed the value of communication to facilitate the activities of the team. The instructor emphasized and appreciated this by verbalizing how difficult can be for the whole team to see how they perform together.

Finally, each member of the team were invited to express personal insights that they identified as good procedures or improvements intended to be utilized in everyday practice. These statements were formulated individually and spontaneously and not imposed by the instructor or other team members. In parallel, during the debriefing, a discussion about medical and caring issues revealed deepened and insightful reflections. One example is an awareness of the need to include the woman in labor in the progress of the delivery, so that a sense of a collaborative effort, “We are doing this together” could be communicated, as well as the importance of communication to create a sense of security for the parents to be. This way, the debriefing sessions also addressed the professional’s experiences from every day practice, which further strengthened the importance and sense of teamwork.

## Discussion

When a delivery is progressing normally, the mother and the midwife create a team. However, if complications occur, the team suddenly grows in number and failure in communication or management could have devastating consequences. This cohort study is comparing clinical outcomes management, staff confidence before and after shoulder dystocia simulations were introduced. The results showed a significant decrease in infants born with a brachial plexus injury after the staff had attended PROBE training. The dominant maneuver to solve the shoulder dystocia changed from posterior arm extraction to internal rotation of the anterior shoulder between the pre and postPROBE groups. In Crofts’ study, posterior arm extraction still dominated after 12 years of training [10], a difference that could be explained by a training program without the hierarchy proposed by HELPPER.

The number of diagnosed shoulder dystocia increased, 0.9/1000 deliveries prePROBE compared with 1.8 and 2.5/1000 in the early and late postPROBE group, respectively. These results are in accordance with findings in the UK, where the proportion was higher but increased over time [10]. There were no differences between the groups concerning potential risk factors for shoulder dystocia such as primiparity, maternal BMI gestational weight gain and diabetes mellitus. During the study period, the number of vaginal deliveries at the unit increased markedly in favor of cesarean sections [24]. The induction rate also increased significantly. This means that a fair amount of women with labor arrest disorders, a well-known risk factor for shoulder dystocia, were delivered vaginally postPROBE compared to prePROBE. This might have contributed to the increased rate of shoulder dystocia. The decreased rate of brachial plexus injury postPROBE persist when relating this complication to all vaginal deliveries which speaks against a higher proportion of milder cases postPROBE. At 6 months follow up, the number of cases with persistent injury was low (one case versus two cases each period) making interpretation difficult. Similar to the present study, no statistically significant reduction in brachial plexus injury at six or 12 months follow up was found in the UK study although numbers declined [10]. Results concerning effects of simulation training on brachial plexus injury at birth differ. In an American setting, the implementation of a shoulder dystocia training protocol significantly reduced obstetric brachial plexus injury [12]. A reduction in brachial plexus injury at birth was also found at a unit with regular multi-professional training on shoulder dystocia management [10]. A cluster randomized controlled trial performed in the Netherlands allocated 24 obstetric units to team training or not and no decreased rate of brachial plexus injury at birth was found in the intervention group [8]. The follow up time in that study was 1 year compared with 8 years in the present study, which might explain the diverting results.

No differences in Apgar score or umbilical artery values among shoulder dystocia cases could be found in the present study, which is in accordance with results presented by Crofts and co-workers [10]. In an Australian study, evaluating the introduction of practical obstetric multi- professional training, an improvement in Apgar score at 1 minute and a decrease in cord lactate was found among all infants born but there were no specific data on shoulder dystocia cases [9]. We also evaluated the rate of maternal complications in terms of anal sphincter injury associated with shoulder dystocia and found no differences between the pre and postPROBE groups. This is in accordance with results from van de Ven et al. who found no difference in risk of anal sphincter injury before and after introducing simulation-based team training [14].

The improved clinical results could apart from a change in shoulder dystocia management, be explained by an increased sense of confidence among staff, feeling safer in handling shoulder dystocia after participation in mandatory reoccurring PROBE training sessions. Another explanation of improvement might be found in the pedagogical practice during the simulation and debriefing session. Observation of how the instructors made use of notetaking during the simulation for commenting and analyzing of the interactions showed how the instructors facilitated individual professional reflections to be relationally linked to the work of the whole team during the simulation exercise. Each performance became in that way connected to a larger context than the individual perspective. The expansion to a larger context was accomplished through instructors commenting on an array of relational interactions, such as between the woman in labor and professionals, in relation to material arrangements of the delivery room, and how these interactions contributed to the delivery process. By keeping a relational focus of the actions in the scenario, the procedure of the whole team became outlined and highlighted against the backdrop of each individual participant. Nyström et al. identified recently two distinct patterns of debriefing sessions, one protocol-based and one more loosely structured collegial conversation [22]. However, neither the imposed structure of the debriefing, nor the lack of structure, ensured that interprofessional collaboration emerged as a salient topic for reflection. The model of facilitating relational reflection adopted in this study seem to provide ways of keeping the collaboration and learning in the interprofessional team clearly focused, and might be one of the factors leading to a successful outcome of simulation as a competence development activity over time.

This study has certain strengths and limitations. A strength is the long-term follow up of a constantly recurring obligatory simulation-based team-training program. Another strength is the different angles from which the effect of the program has been evaluated, including maternal and infant outcome data, management data, staff confidence follow up and scrutinizing the scenario debriefing process from a learning perspective. Available data on deliveries over all made it possible to present rates of shoulder dystocia as well as brachial plexus injury related to number of births, both overall and among vaginal deliveries before and after introduction of PROBE. There has been no change in the management of shoulder dystocia (HELPERR) after the introduction of PROBE and the shoulder dystocia scenario has been part of every PROBE training event since it started. A putative important factor of adherence to PROBE training might have been that midwifes and doctors were scheduled for PROBE immediately from introduction without having any other tasks planned for these 3 hours as an integral part of their clinical work.

## Conclusions

In conclusion this study showed that to introduce and sustain a shoulder dystocia training program for delivery staff increase the number of infants born after a diagnosed shoulder dystocia without a brachial plexus injury or fracture of the clavicle or humerus. The staff confidence on handling shoulder dystocia in daily clinical care increased after PROBE training. The analysis of the qualitative data showed that the main learning effect in the debriefing model used was that discussions concerned the team and related to daily clinical practice.

## Additional file

Additional file 1: Staff questionnaire. (DOCX 21 kb)

## Abbreviations

ALSO®: Advanced Life Support in Obstetrics
BMI: Body mass index
HELPERR: H— call for Help, E—consider Episiotomy, Legs—McRobert’s maneuver, Pressure—Suprapubic pressure externally, Enter—Enter the vagina using internal pressure to reduce impacted shoulder, finally using a Wood’s screw maneuver to bring the shoulders into oblique diameter and 180 degrees rotation, and, if necessary, Remove—the posterior arm and the last R—Rotate the patient to her hands and knees
PROBE: Practical Obstetric team-training
SEM: Standard error of the mean
