# Supplementary material for: Ten years of simulation-based shoulder dystocia training- impact on obstetric outcome, clinical management, staff confidence, and the pedagogical practice - a time series study
Source: BMC Pregnancy Childbirth. 2018 Sep 5;18:361. doi: 10.1186/s12884-018-2001-0 (PMC6125924; doi:10.1186/s12884-018-2001-0)
Supplement: Supplementary file 1 — Staff questionnaire. (DOCX 21 kb) [file 12884_2018_2001_MOESM1_ESM.docx]

Questionnaire answered in a web survey using the survey tool LEO.

| **1. Have you attended PROBE?** |
| --- |
| Yes |
| No |
|  |
| **2. Do you feel more confident in your daily practice managing an obstetric emergency situation after PROBE training**? |
| Yes |
| No |
|  |

| **3. How has PROBE training affected the communication in a real emergency situation?** |
| --- |
| comments |
|  |
| **4. How has PROBE training affected the team work in a real emergency situation?** |
| comments |
|  |

| **5. Do you feel more confident in your daily practice managing postpartum hemorrhage after PROBE training?** |
| --- |
| Yes |
| No |
| I have never been in the situation |
|  |
| **6. Do you feel more confident in your daily practice managing vacuum extraction after PROBE training?** |
| Yes |
| No |
| I have never been in the situation |
|  |

| **7. Do you feel more confident in your daily practice managing a shoulder dystocia after PROBE training?** |
| --- |
| Yes |
| No |
| I have never been in the situation |
|  |
| **8. Do you feel more confident in your daily practice managing a twin delivery after PROBE training?** |
| Yes |
| No |
| I have never been in the situation |
|  |

| **9. Do you feel more confident in your daily practice managing a breech delivery after PROBE training?** |
| --- |
| Yes |
| No |
| I have never been in the situation |
|  |
| **10. Comments:** |
|  |
